# Caring for pregnant refugee women in a turbulent policy landscape: perspectives of health care professionals in Calgary, Alberta

**DOI:** 10.1186/s12939-018-0801-5

**Published:** 2018-06-26

**Authors:** Anika Winn, Erin Hetherington, Suzanne Tough

**Affiliations:** 1 Department of Health and Science Education, Cumming School of Medicine, University of Calgary, Calgary, AB Canada; 2 Department of Community Health Sciences, Cumming School of Medicine, University of Calgary, Calgary, AB Canada; 3 Department of Pediatrics, Cumming School of Medicine, University of Calgary, Calgary, AB Canada; 4 Owerko Centre, Child Development Centre, #355, 3820- 24 Avenue NW, Calgary, AB T3B2X9 Canada

**Keywords:** Refugees, Pregnancy, Pregnant women, Delivery of health care, Prenatal care, Health care providers

## Abstract

**Background:**

Female refugees can be a vulnerable population, often having suffered through traumatic events that pose risks to their health, especially during pregnancy. Pregnancy can be an entry point into the health care system, providing health care professionals the opportunity to gain women’s trust, connect refugees with resources, and optimize the health of mother and child. Policies surrounding the provision and funding of health care services to refugees can impact access to and quality of care. The aim of our study was to understand the experiences of health care professionals caring for pregnant refugee women in Calgary, AB, taking into consideration recent contextual changes to the refugee landscape in Canada.

**Methods:**

We conducted ten semi-structured interviews with health care professionals who provided regular care for pregnant refugee women at a refugee health clinic and major hospital in Calgary, Alberta. Interviews were recorded, transcribed, and analyzed using an interpretive description methodology.

**Results:**

Health care providers described several barriers when caring for pregnant refugees, including language barriers, difficulty navigating the health care system, and cultural barriers such as managing traditional gender dynamics, only wanting a female provider and differences in medical practices. Providers managed these barriers through strategies including using a team-based approach to care, coordinating the patient’s care with other services, and addressing both the medical and social needs of the patient. The federal funding cuts added additional challenges, as many refugees were left without adequate health coverage and the system was complicated to understand. Health care providers developed creative strategies to maximize coverage for their patients including paying out of pocket or relying on donations to care for uninsured refugees. Finally, the recent Syrian refugee influx has increased the demand on service providers and further strained already limited resources.

**Conclusion:**

Health care providers caring for pregnant refugee women faced complex cultural and system-level barriers, and used multiple strategies to address these barriers. Additional system strains add extra pressure on health care professionals, requiring them to quickly adjust and accommodate for new demands.

**Electronic supplementary material:**

The online version of this article (10.1186/s12939-018-0801-5) contains supplementary material, which is available to authorized users.

## Background

In 2018, the number of forcibly displaced people is at a record high, with the United Nations Higher Commission for Refugees calling for a coordinated global response to resettle such vast numbers of refugees [1]. Refugees are defined according to the 1951 Refugee Convention, as “individuals who flee their country of origin owing to a well-founded fear of being persecuted for multiple reasons, and believe that they cannot safely return to that country” [2]. Canada has a long tradition of participating in global humanitarian actions, and in 2016, admitted 46,700 refugees into the country, which is the largest number of refugees resettled into Canada in a single year in nearly four decades [3]. The population profile of refugees in Canada changes annually, but in 2016, the top five countries of refugee origin were Syria (33,266), Eritrea (3934), Iraq (1650), the Democratic Republic of Congo (1644) and Afghanistan (1354) [4].

When humanitarian crisis hits, women and girls tend to bear the heavier burden of caring for both families and communities at large, often experiencing pre-migratory loss of family members and long family separations [5]. Female refugees are at a higher risk of experiencing violence, sexual abuse, exploitation, as well as suffering from isolation and loss of existing social support systems, which may affect their health during pregnancy [5–7].

Caring for pregnant refugee women differs from caring for Canadian-born pregnant women, as there are a multitude of barriers that health care professionals must manage [8]. Given their experiences, refugee women may distrust care providers, be resistant to North American healthcare practices and procedures, and face communication barriers with their providers [7, 8]. However, pregnancy, and corresponding prenatal care can be an entry point into the health care system for refugee women. This provides the opportunity to gain women’s trust with the health care system, mitigate concerns or fears, and encourage regular use of preventive health care services to maintain their and their children’s health [7, 9]. Furthermore, prenatal care providers also play an important role in connecting women with social services, prenatal groups and other support groups during pregnancy [10].

The provision of health care coverage for refugees in Canada has been through several changes in recent years, further complicating the delivery of health care for refugees. Prior to June 30th of 2012, all refugees received temporary limited health care coverage from the Interim Federal Health Program (IFHP), until they were able to qualify for provincial health care [11]. However, on June 30th, 2012, the Conservative Government of Canada implemented severe funding cuts to the IFHP [12, 13]. The funding cuts affected the coverage of health services for most refugee groups, and what services were covered for which groups were constantly changing. Prenatal and child care in particular, were affected. Finally, on April 1st of 2016, the newly-elected Liberal Government of Canada announced that they would be restoring funding to the IFHP to pre-2012 levels [11–13]. Overall, the several changes to the IFHP in Canada over a short span of time caused extreme confusion amongst health care professionals and created additional barriers to providing care to a group of already vulnerable individuals [11–13]. Table 1 provides a summary of the health care coverage for different classifications overtime.

**Table 1:** Summary of Health care coverage for refugees

Refugee Classification	Description	IFHP Coverage Pre-2012	IFHP Coverage 2012–2016
Government Assisted Refugees	Sponsored by the Government of Canada (up to one year)	Basic, supplemental and prescription drug services split between the IFHP and provincial health care coverage	Same as pre-2012 coverage
Privately Sponsored Refugees	Sponsored by private citizens/ groups (up to three years)		IFHP covered basic services; lost supplemental and prescription drug coverage
Claimant Refugees “Safe” Countries	Seeking protection from a Canadian Immigration Officer from a country designated as “safe”, such as Croatia, Mexico and Chile	Basic, supplemental and prescription drug services covered solely by the IFHP, until claimant receives permanent residency and qualifies for provincial health care coverage	Lost all coverage, unless health condition was considered a public health threat
Claimant Refugees “Non-Safe” Countries	Seeking protection from a Canadian Immigration Officer from a country designated as “not safe”, such as Somalia, Iraq and Lebanon		IFHP covered basic services; lost supplemental and prescription drug coverage
Rejected Claimants	Refused refugee status in Canada and must take alternative actions to stay in Canada	Not covered for any health services	Not covered for any health services

Now in its 7th year, the Syrian war is being considered one of the worst humanitarian crises of all time, producing an estimated 4,812,204 million refugees [14]. The Government of Canada announced in November of 2015 that they were going to admit 25,000 Syrian refugees into Canada by the end of February 2016, a goal which they surpassed [15]. As of January 29th, 2017, 40,081 Syrian refugees have been resettled into 350 communities across Canada, resulting in a substantial increase in the number of refugees requiring access to health care services [15]. Syrian refugees as a population in themselves, are a distinct group of refugees. It is estimated that one third of all Syrian refugees coming to Canada are women of childbearing age [16, 17]. The majority of Syrian refugees speak Arabic, one of the most common immigrant languages in major Canadian cities [18]. Additionally, Syria was once a middle-income country with well-established education and health care systems [17]. These factors, along with the Syrians being resettled into Canadian communities in large groups, have the potential to make the resettlement process into Canadian society easier for Syrian refugees. Therefore, the experience of providing health care to Syrian refugees may be different than that of the general population of refugees.

Most previous literature regarding pregnant refugees in Canada examines barriers to care faced by pregnant refugees or highlights the increased levels of pregnancy complications and poor birth outcomes faced by refugees including increased c-section rates, higher levels of low birthweight babies and others [19–23]. These studies suggest possible reasons for poorer outcomes, but there is no direct evidence of perspectives of health care providers as to systemic barriers facing access to care. This may be due to a variety of reasons, including refugees being a vulnerable population to access, and that every region in Canada has a different strategy for caring for refugees [24]. The majority of cities have opted for a care-model of matching refugees to a primary care clinic in the city in which they are resettled. In cities with these models of care, physicians would likely not have extensive experience working with refugees. There are few cities in Canada, including Calgary, Hamilton and Kitchener, which have designated refugee health clinics. Furthermore, previous studies tend to combine immigrant and refugee populations when studying prenatal care, failing to distinguish between the two [24]. Given the circumstances that refugees go through to resettle in Canada, as well as the differences in their provision of healthcare through IFHP, refugee populations cannot be treated the same as immigrant populations. Doing so would diminish the extraordinary experiences and challenges that female refugees especially, often face [10, 24, 25]. Therefore, research on the experience of health care professionals caring for pregnant refugee women, could inform care guidelines. This information would be timely given the recent changes in health funding and the ongoing recruitment of refugees into Canada.

The aim of this study was to understand the experiences of health care professionals providing care to pregnant refugee women in Calgary, Alberta. This study had three specific research objectives:

1. What barriers and facilitators do health care professionals experience when caring for pregnant refugee women?
2. How have the changes to the IFHP between June 2012–April 2016, affected health care professionals provision of care?
3. How has the Syrian Refugee crisis, and subsequent influx of refugees into Calgary (November 2015- present), affected health care professionals provision of care?

## Methods

### Theoretical framing

Our study draws on theoretical approaches based in human rights, specifically the right to health. Canada is a signatory to the International Covenant on Economic, Social and Cultural Rights, meaning that as a country, they are obliged to take steps to protect the rights enshrined in the Covenant [26]. Article 12 recognizes the “right of everyone to the enjoyment of the highest attainable standard of physical and mental health”. This includes the right of refugees to have access to medical services, and for provisions to reduce neo-natal mortality and morbidity and contribute to healthy child development [26].

Further, the United Nations have published several documents with the intent to reduce disparities in health between the host and refugee populations, ensure that refugees have the right to health and do not feel stigmatized when accessing health services, and to minimize the negative impact of migration on health [27–29]. They emphasize responding to the global refugee crisis through evidence-based strategies. As a global community, there must be a focus on empowering all stakeholders involved in protecting refugees, such as health care professionals, to maximize their capacity to provide positive contributions, and to work towards solving problems and addressing barriers that limit refugee’s livelihood [27–29]. The Covenant and principles articulated in the United Nations documents remained central to our research, with the overall goal of contributing to the body of knowledge to improve the wellbeing of female refugees migrating to Canada.

### Methodology

The methodology followed for this study was interpretative description, a qualitative method developed specifically for understanding knowledge pertaining to clinical contexts [30]. Interpretative description posits that reality is complex, constructed, contextual and subjective, that the knower and what is known are inseparable, and that no *apriori* theory could encompass the realities within a specific context [30]. Interpretive description advocates for inductive, creative and flexible data analysis in order to generate knowledge and provide clinical recommendations specific to context of the study [30].

The emphasis on the role of the unique clinical setting in generating knowledge was critical in our decision to follow the interpretive description methodology. We needed to account for the fact that the provision of health care for refugees differs across Canada, and that we were focused on specifically understanding the experiences of health care professionals caring for pregnant refugee women, a sub-set of the entire refugee population. Interpretive description allowed us to conduct a study that contributed to the overall body of knowledge on the provision of health care to refugees in Canada, while still emphasizing and accounting for the specificity of our study results to the Calgary context.

### Study setting

We non-randomly identified the Mosaic Refugee Health Clinic (MRHC) as an ideal location to conduct interviews. The MRHC is one of the only specialized refugee health clinics in Canada and utilizes a central and comprehensive model for providing services for refugees that addresses their physical, psychological and social needs, and helps to ease their settlement into their new city [31]. The MRHC consists of an interdisciplinary team of health care professionals, including family physicians, obstetricians and gynaecologists, pediatricians, internal medicine specialists, chronic and infectious disease nurses, psychologists, social workers and specially trained front-desk staff. All staff at the MRHC are extensively trained in refugee health and tropical diseases, are aware of the different classifications of refugees and subsequent variances in health care coverage and are familiar with the barriers to health care and social services that refugees face.

The clinic is particularly unique in that approximately 90% of all refugees who come to Calgary will receive primary health care at the MRHC for their first two years post-arrival [31]. When the two-years is up, or the MRHC feels the patient is ready to be transitioned out the clinic, they will help to connect the patient to a family physician within their community [31]. The clinic provides primary and multi-disciplinary care services, such as: women’s health services including pregnancy and postpartum care and family planning; family health services including tropical disease care, health education and help accessing social services and interpreters, and; mental health care [31]. The specialized nature of the clinic has been successful in quickly responding and adapting to contextual factors that affect refugee health and being a resource for other health care professionals and social services in Calgary to connect refugees new to the city to.

### Study design

We chose a qualitative study design focused on obtaining insight from experts most knowledgeable in the systemic and policy influences on perinatal refugee health. We chose to focus on health care professionals so that we could address systemic and contextual factors (ex; funding changes, increase in service demands, etc.) that impact the provision of health care services, as perceived by health care professionals themselves. Moreover, we deliberately chose not to directly interview refugee women for two reasons. First, most would have had a single contact with the health care system, and would therefore be unable to discuss the changes to the system over time. Second, refugees are a vulnerable population, many of whom have experienced significant trauma during their recent past. In medical research, it is important not only to minimize burden but maximize benefit to the subject population. [32]. We felt interviewing refugees directly could place undue burden on them, with minimal chance of any direct benefit.

### Study sample

We conducted semi-structured in-depth interviews with health care professionals who have regular experience providing care to pregnant refugee women in Calgary. All interviews were conducted by AW and were 30–60 min in length. AW received training in qualitative interviewing and conducted several pilot interviews to refine her skills and clarify the interview guide.

The inclusion criteria for selecting our sample was as follows:

i. Physicians, obstetricians and gynecologists, midwives, registered nurses, social workers, or any other health care professionals who have experience providing care for pregnant refugee women between the period of 2012–2016; AND
ii. Works at the MRHC or another medical centre in Calgary, Alberta; AND
iii. Is fluent in English

The exclusion criteria for selecting our sample was as follows:

i. Health care professionals who do not have recent experience (2012–2016) caring for pregnant refugee women at a health care setting in Calgary, Alberta

Participants were recruited through purposive snowball sampling. We developed a relationship with the director of the MRHC, who connected us with a variety of health care professionals at the clinic who met the inclusion criteria for our study. All participants interviewed at the MRHC were further asked to identify other potential participants with the necessary experience, in order to expand our study reach, and reduce the risk of bias by having only one individual identify participants. Two participants subsequently connected us with the antepartum, labour and delivery, and postpartum unit of a major hospital located in a part of Calgary attending to the needs of immigrant and refugee populations.

Our final study sample consisted of ten participants: eight from the MRHC and two from the hospital. The final sample included a variety of health care professions: 3 family physicians, 2 low-risk maternity care physicians, 1 chronic disease nurse, 2 labour and delivery nurses, and 2 social workers. All of our participants were female, 1 participant was foreign-born, 3 participants were visible minorities, and years of experience ranged from participants being in their first 5 years of practice to having more than 15 years of experience. Since the MRHC is a small clinic where participants are easily identifiable, we have chosen not to provide a more detailed description of our study sample so to protect the anonymity of our participants.

### Data analysis

All interviews were recorded upon participant consent and transcribed. Our data analysis was an inductive and iterative multi-stage process, completed using NVivo 11 (QSR International, Cambridge, MA) Qualitative Data Management Software for Coding and Analysis. The first two authors developed the coding framework over four rounds of independently coding individual interviews, meeting to compare and discuss codes, and then revising the coding framework. The final coding framework consisted of first level, second level and third level themes. Both authors then independently re-coded line-by-line, all interviews using the final coding framework. Subsequently five overarching themes were developed.

### Quality assurance

We took several measures to improve the quality of the study. Two researchers were involved in developing the coding framework and overarching themes in order to minimize bias in interpretation of results. An auditing approach was adopted and records of all decisions during each stage of the research process was documented. As a measure of triangulation, we interviewed different professionals to obtain different viewpoints. However, our overall goal was to develop overall themes and not a comparison by profession. Member checking was deployed to validate our study findings. All participants were sent a report of our findings, along with their individual quotes, and asked to reflect on whether we appropriately captured their intentions. Additionally, we delivered an oral presentation to the entire staff at the MRHC (including both participants and non-participants). We asked for feedback following the presentation, and all attendees reflected that they agreed with our findings and would not add anything additional. From this feedback, we believe that we achieved thematic saturation of our results specific to the MRHC. At the presentation we also worked with the staff members of the MRHC to develop and refine the recommendations presented in our discussion.

Finally, this study conforms to the BioMed Central Qualitative research review guidelines – RATS. Ethical approval was granted for this study by the University of Calgary Conjoint Health Research Ethics Board (CHREB). The Ethics identification number is REB16–1488.

### Results

Overall, health care providers spoke to the general barriers and facilitators in providing care for pregnant refugee women. They identified multiple barriers facing this heterogeneous group, and engaged in multiple strategies to address these barriers. Both the period of funding cuts, and the Syrian refugee crisis added additional barriers and health care providers engaged in additional strategies to address these barriers. Participants who did not have specialized refugee health training were uninformed about caring for refugees and did not engage in specific strategies for this population.

**Fig. 1:**
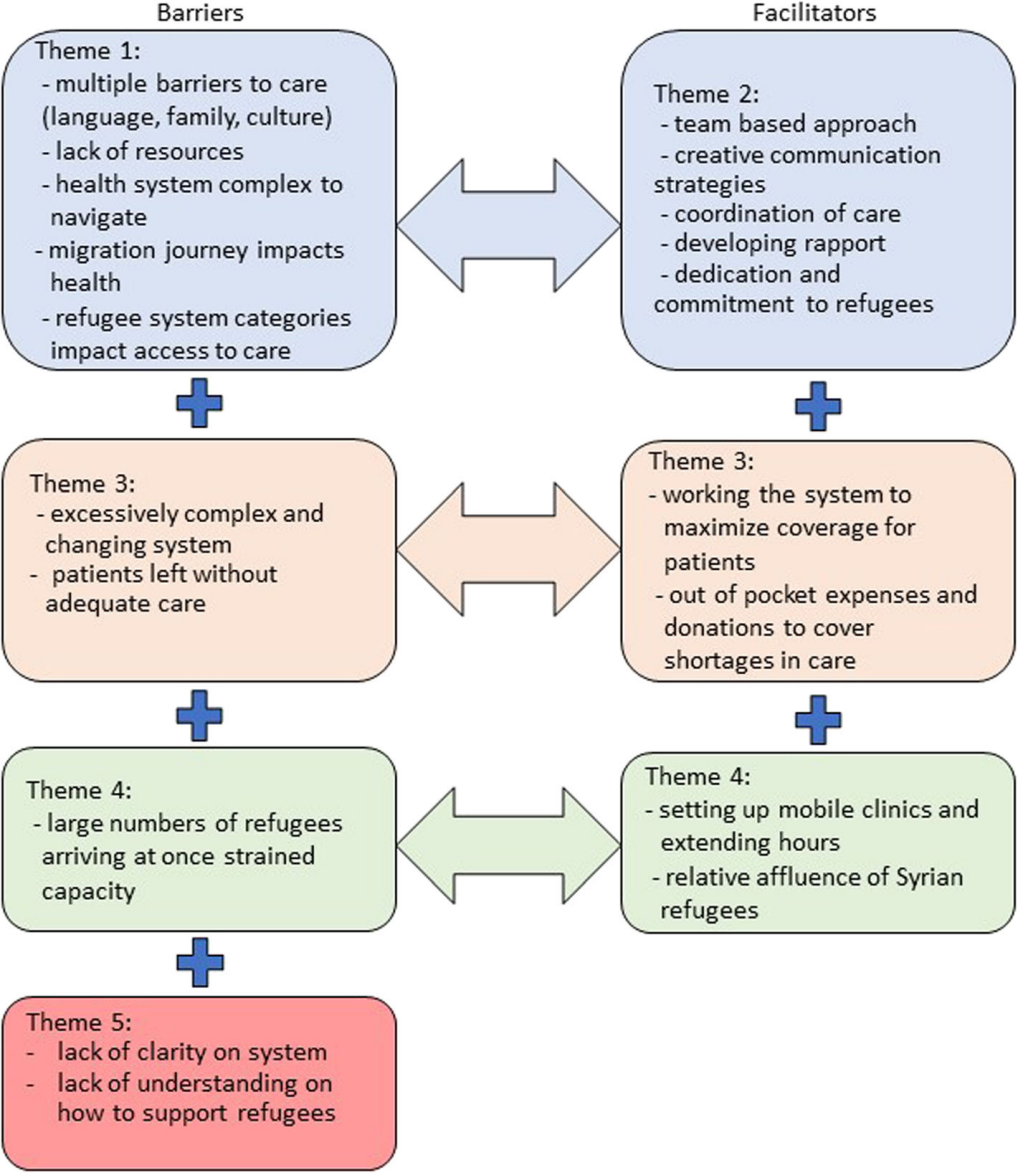
Synthesis of barriers and facilitators to caring for pregnant refugee women

Our results have been structured under five overarching themes with explanations and illustrative quotes. The first two themes speak specifically to the first research objective, and the third and fourth themes address the second and third research objectives, respectively. Finally, a fifth theme developed out of the health care professionals at the hospital being unable to answer many of our interview questions. The aim of our analysis was to provide a synthesis of the perspectives of the health care professionals we interviewed, and not to conduct inter-group comparisons. We opted not to identify the participants by occupation, as this would make them easily identifiable. Figure 1 provides a visual depiction of the relationship between the five themes.

### Theme 1: Pregnant refugees are a heterogeneous population facing multiple barriers to care

Pregnant refugee women are a diverse and vulnerable population that face multiple and complex systematic barriers to perinatal care. A full list of barriers can be seen in Additional file 1 which contains the overall coding framework for the study. The barriers described in detail include: language and cultural barriers, lack of resources, difficulty navigating a complex system and diverse migratory experience and legal status.

First, language and patient comprehension barriers were challenging, especially in acute care settings where it could be difficult to ensure that women understood what was happening and were providing informed consent for procedures.

*“P*_*4*_ *: When you’re in the delivery and there’s an acute situation, and you’ve got to do a vacuum or the obstetrics, the obstetrician has to come in […], sometimes there’s not time to go get the language line phone, and then be put on hold, having to have a back and forth conversation translated, back to do you understand what the risks are. So, that’s one of the barriers, it is the language in acute care.”*

Second, health care professionals mentioned a lack of resources for pregnant refugee patient’s access in Calgary. Notably, there are few organizations which provide tangible support for expecting mothers and the majority of available external resources have limited access to language support services.

“*P*_*3*_*: We need access to more resources in this city […], and by resources sometimes its material goods. Like, they don’t have to have everything they need for baby, but just like a start-up, sometimes those baby boxes that I know were in the media for a while. And then also in specific communities […] I think just places where women can get together and meet.”*

Third, participants said that the health care system was complicated for patients to navigate, and that cultural specific expectations and norms, such as traditional gender roles and women not agreeing with induction of labour, caesarean deliveries, or the use of epidurals, often had to be managed.

*“P*_*5*_ *: I know how distressing it is for them [refugees] to be in a new country, not understanding the language, not understanding the culture and the policy, having to come for so many medical and pregnancy related appointments, and sometimes they ask the question why? Why the blood works, why the testing, why the ultrasounds, why they make me come back every month, and then twice a month, and then every week in the last trimester?”*

*“P*_*2*_ *: I think probably the biggest challenge is no male physician for the delivery, and we cannot guarantee that. It’s not the way it works in Canada [...] if she goes into hospital and there’s an emergency, it may be a male OB doing the C-Section or you know, helping out with the shoulder dystocia right or whatever it might be.”*

Fourth, health care professionals explained the importance of considering the unique background of each patients and the migration journey they took to arrive in Canada and their legal status upon arrival. Knowing the migration journey of the refugees is also necessary to provide a thorough medical investigation of each patient, especially if the patient arrives in Canada pregnant or quickly becomes pregnant upon arriving. Overall, health care professionals considered patients arriving in Calgary already pregnant, to be of elevated-risk and more stressful to manage, because of unknowns surrounding the pregnancy.

*“P*_*1*_ *: I’ve seen everything from people presenting and not knowing they were pregnant, and umm all the way up to people who walk into the clinic ready to deliver […] I’ve met people on the elevator and done an initial check and then sent them on to the hospital to have their baby.”*

Finally, the category of refugee that an individual is admitted into Canada affects the services, resources and supports they have access to. These factors influence how refugees resettle in Canada, interact with the health care system, as well as women’s expectations during her pregnancy.

*“P3: Those who come as claimants have a lot more barriers I think in Canada than you know private and government sponsored [refugees].”*

### Theme 2: Health care professionals specialized in refugee health engage in diverse strategies of care

Health care professionals at the MRHC engaged in diverse and creative strategies to optimize the care of their patients, given the unique interdisciplinary structure of the clinic and health care professional’s expert knowledge of refugee health. These strategies include providing a team-based approach, creative communication, coordination of care, building rapport, and an overall dedication to the unique nature of caring for refugees.

First, health care professionals noted that a team-based approach to care was critical to the quality of care patients receive.

“*P*_*8*_*: Mosaic is extremely well supported with other disciplines. So, we work closely with the social workers and they’re very instrumental in helping provide supports, just resources, physical resources, but also trying to get the social supports in place to.”*

Second, health care professionals emphasized the importance of deliberate and clear communication with patients. They have to be constantly alert that their patient may not understand what is going on, so it is up to them to take the extra time to ensure the patient’s comprehension. This involved being creative in how they present information to patients and trying new strategies, such as getting patients to repeat all information back to them, drawing maps and utilizing visual aids.

*P5: “I always use highlighters, highlight the relevant information, and then print a navigation map, what bus you take, where do you get off. I circle everything, I’m always telling them if you are ever lost you just show this to the driver, they will know exactly where to tell you to get off. When I’m calling the agencies, I’m preparing them to make sure they know my patient doesn’t speak the language. [I ask] do you have anybody in the agency who speaks that particular language, any volunteer, anybody? If not, I’m asking [the refugee], please bring your interpreter with you if you can, and I’m always trying to tell them, bring an adult interpreter. We do not want for children to be exposed to that you know, taking on the families problems, because perhaps it becomes heavy for them.”*

Third, a large component of patient-care provider communication was coordinating the care of their pregnant patients, both within and outside of the clinic. Health care professionals said that they coordinate lab appointments, ultrasounds, and specialist appointments, as well as follow up with patients after each appointment to ensure that information was correctly conveyed to them.

*“P*_*6*_ *: We coordinate appointments, lab appointments, ultrasounds, other specialist appointments, dentist appointments, then we rebook them, […] and we also make sure that other specialists recommendations are followed up with them as well and coordinating that, if that needs coordinating.”*

*“P*_*2*_ : If it was me as the pregnant women [at the ultrasound clinic], they [the radiologist] would have said this is the issue, we need to book you here and you need to then go here, and that would have been the end of it. But instead, the [refugee] patient doesn’t understand […] [Mosaic] eventually gets the report, we’re like sh*t did she, like, go to this? We follow up with people like all the time. No, she didn’t go to it. We have to rebook it, then we have to call her in here to explain exactly what’s been going on and what the procedure is [….] it’s just a lot of logistical work”.

Fourth, health care professionals commonly mentioned that the beauty of working with pregnant women was that it allowed them the time to develop a rapport with their patients. The continuity of prenatal care provided health care professionals the opportunity to provide care tailored to the patient’s unique needs, addressing both their medical and social wellbeing. The MRHC tries to ensure that all pregnant women are seen by a social worker at least once during their pregnancy to help address the often-unmet social needs of expectant refugee mothers.

*“P*_*7*_ *: Not a lot of [health care professionals] take the extra step to look at what’s going to happen when the baby’s born […] sometimes you need the physician who’s the first point of care often for the patients, to recognize their social concerns in terms of you know social determinants of health […] so that you can refer her to the proper resources because she is so vulnerable, and it’s really common that these women don’t get any services.”*

Finally, the health care providers exhibited certain characteristics which exemplified their dedication and commitment to caring for refugees. They described themselves as advocates for the refugee population. Our participants emphasized the importance of social justice and raising awareness to the challenges and systematic barriers that this already vulnerable population face.

*“P*_*5*_ *: I should say 75% of all my job is advocacy. Opening and pushing the doors, sending the letters, sending the fax, calling them, pushing them, leaving them messages, in order to be the speakers of those [refugees] who have no voice.”*

In general, we observed that health care professionals at the MRHC were highly committed to the population they worked with and seemingly goes above and beyond basic standards of care to optimize the wellbeing of their patients. These qualities have been positively reflected upon by patients of the MRHC.

*“P*_*3*_ *: There’s recently some feedback about what our patients like about our clinic the most, and the first was that we treat everyone with dignity and respect, and second was access to the social work supports […] we are people’s families here, […] Everybody who works here [at the MRHC] are very passionate about working with refugee populations and then that shows right.”*

### Theme 3: Funding cuts created a confusing system which jeopardized care

The refugee landscape in Canada has undergone changes over the last several years, which has resulted in additional strains on the health care system, magnifying existing problems, and requiring care providers to go to exceptional lengths to ensure quality of care. Health care providers noted that these cuts played out in two critical ways. First, health care providers describe the funding cuts creating an excessively complex system which left some patient without coverage. To address this they became system experts and patient advocates and paying out of pocket to cover expenses. Second, due to the cuts, some patients were left without any coverage, and the MRHC created a donation system or covered expenses out of pocket to ensure quality of care.

First, health care professionals described understanding the health care coverage for refugees and the system in general during the period of the cuts, as being extremely complicated and confusing. Coverage levels for different groups of refugees kept changing, government agencies were not current on policy changes, nor was the online federal health care portal. Health care professionals at the MRHC said that it was up to them to determine the level of coverage, to ensure their patients received the maximum coverage they were eligible for, which was described as being exhausting. Overall, this was a period marked by a lack of clarity and confusion, and health care providers expressed extreme frustration with the system.

*“P*_*2*_ *: We heavily involved like, social work to figure it out [levels of coverage], like all of the front staff, and they had to be like on the ball. And then things kept on changing […] so it just made it very confusing […] Yeah, so it was just constant energy, umm involved in this.”*

In order to address the complex and changing system, health care professionals at the MRHC said that they ‘learned the ins and outs’ of the health care system, so they could obtain maximum coverage for their patients. This included applying for benefit programs, communicating through email and phone call to the ‘right’ people, and being creative in ordering clinic supplies. Essentially, health care professionals described going to extra lengths to try and accommodate their client’s needs. Additionally, during the cuts, health care professionals said that they would pay out of pocket to cover the health care services and medication for pregnant refugee patients without adequate health care coverage.

*“P*_*4*_ *: In the past, a small number of our refugee patients (refugee claimants) did not have any health care coverage. Our clinics never turned them away. They don’t have the money, so what do you do? You have to be a Canadian, just be a Canadian.”*

Second, during the 2012–2016 federal funding cuts to refugee health care, some patients being left with inadequate health care coverage. Health care professionals described that the health of mothers and Canadian-born children were put at risk during this period, as many expectant mothers were not covered for comprehensive perinatal care.

*“P*_*1*_ *: Claimants had nothing […] which was ridiculous because then these kids are going to be born, they are going to be Canadian, and them having poor prenatal outcomes was to no benefit to anyone.”*

*“P*_*2*_ *: Some did have their kids in the NICU and one even had their child die, and then and they ended up being presented with a massive bill, massive bill. So, it’s just you know, it’s tragic on a personal level, and then to have the added financial burden on top of it, it was cruel. And the babies are Canadian, if they’re born here, they’re born as Canadians.”*

To offset the cost of services that the MRHC provided during the period of the funding cuts, the clinic established a donation fund to cover costs for some of the uncovered services for refugee patients, with maternity care being a priority. Health care professionals described this as an extremely expensive period for service providers in Calgary.

*“P*_*1*_ *: Thankfully, we have a private donation account that we have anonymous donors donating too, as well as all of the docs that work at the MRHC, any teaching that we do, like that we’re paid by the university, we put all of the stipends […] into our donation account.”*

### Theme 4: Syrian influx created additional strains on existing problems

Health care providers described the Syrian refugee influx as a challenging situation with unique features. The arrival of large number of refugees created system strains as a result of the increased number of refugees requiring care. However, the Syrian refugees had some advantages in managing the transition to Canada compared to other refugee groups due to the relative level of economic development in Syria before the war, and the publicity generated by the government’s public commitment to refugees.

First, health care professionals at the MRHC said that large numbers of Syrian refugees would arrive in waves, and created additional demands on care without additional system level support. The clinic deployed several strategies include hiring more staff, extending clinic hours, partnering with other primary care clinics in Calgary, and operating out of makeshift clinics located at hotels where the Syrian refugees were staying when they arrived in Canada. There was an increase in the number of refugee women arriving in Calgary already pregnant, or becoming pregnant quickly upon arrival. Those who arrive pregnant created additional stress because they require immediate and ongoing prenatal care.

“*P*_*6*_*: So, we had a walk-in in clinic at the Travelodge and I was one of the [health care workers] mandated at the walk-in clinic […] and if they came to me and they were prenatal, I would definitely call the clinic that day and say we need an appointment for this prenatal patient, can we fit her in? Literally fit her in. So, then we would require some rearranging of appointments, and scheduling and all that stuff, yeah, yeah, just because we wanted to confirm how far along they were and get everything kind of set up for her.”*

*“P*_*2*_ *: It’s been super busy […] the Syrians doubled our numbers, so we’re completely maxed out. Lots of pregnant women within the Syrian Refugee community.”*

Second, health care professionals described the Syrian refugees as low risk, literate, familiar with prenatal care and able to communicate in Arabic. These factors provide Syrian refugees with greater opportunities to form support networks and access resources in Calgary, compared to other groups of refugees. Additionally, the Syrian refugee crisis was highly publicized and Syrian refugees received special funding from the Canadian government and attention from the media. These factors helped to alleviate some of the needs of the Syrian refugee patients requiring care.

*“P*_*4*_ *: The Syrians are interesting because compared to the rest of the people that we’ve ever had, they’re actually fairly well educated and they’re actually not umm, they haven’t their whole life been […] disadvantaged or a refugee.”*

*“P*_*2*_ *: So, Syria is a middle-income country, Damascus is very similar to Calgary in many ways, so is Aleppo. Like a lot of people from the cities anyways have had really good education, most have had access to good public health programming. They speak Arabic, which is very commonly spoken in Canada, especially amongst healthcare providers. They’re a little better able to function in Calgary then perhaps our Somali refugee who spent 20 or 30 years in a refugee camp.”*

### Theme 5: Health care professionals unfamiliar with refugee health may be overwhelmed

The two participants from the major hospital were unable to respond to questions regarding system challenges for refugees. For example, both participants were unaware that refugee health care is provided through federal funding, and that over recent years there has been changes to that funding resulting in the loss of health care coverage for refugee patients.

*“P*_*10*_ *: I can’t speak to that [the funding cuts] because I’m not in that position, I don’t have control of the funding but like so no, I can’t speak to that.”*

As was explained to us, nurses in Canada do not receive any training regarding different types of health care coverage. However, one nurse expressed a desire to receive such training to improve the quality of care she is able to provide.

*“P*_*9*_ *: Sometimes our [refugee] patients even ask us in triage like financial concerns, and I don’t know what to say at all. Like that’s something I would like to be more educated on, like what kind of services are available to you [refugees].”*

Notably, four health care professionals commented that they were unfamiliar with the special needs of refugee patients and systematic challenges until they began working at the MRHC. Two participants mentioned that they were only familiar with refugee populations because they had completed medical school rotations and fellowships at the MRHC, and another two participants had prior experience volunteering with refugees. Three participants also commented that while their previous experience working with immigrants contributed to their understanding of refugees, working with refugees is itself a completely different experience.

*“P*_*2*_ *: I worked at urgent care [before working at the MRHC] and I don’t think I even inquired [if a patient was a refugee], like I wouldn’t have even known they were refugees or not.”*

### Synthesis

Overall, refugees are a distinct and vulnerable population to care for. Refugees and their care provider’s face numerous systematic barriers to health care, and health care professionals must also accommodate for additional barriers to care, such as language, comprehension, and pregnancy expectations and norms specific to a patient’s cultural background. The unique nature of the MRHC allowed them to utilize a range of tools to provide comprehensive patient care addressing physical, psychological and social concerns. Additionally, contextual factors such as the IFHP funding cuts and Syrian refugee influx, created additional strains on an already overburdened and under-resourced system. These factors required an in-depth understanding of the impact of these changes, and additional strategies to be deployed to continue providing high level care to the refugee population. The MRHC was able to accommodate for these dynamic and overwhelming system demands, on top of managing the basic challenges and barriers to health care that refugees regularly experience. However, health care professionals not familiar with refugees may struggle to identify and understand the special needs of refugee patients and systemic barriers they experience, or have the resources to adequately address them.

## Discussion

Our study provides insight into the unique challenges faced by health care providers caring for pregnant refugee women in a turbulent political landscape. Overall, we found that pregnant refugees face unique challenges, that were increased by funding cuts and the arrival of large numbers of refugees at once. The centralized model of the MRHC was able to respond to the complex needs of refugees and adapt to this shifting landscape creatively in order to ensure quality of care to women, but those unfamiliar with the specific needs of refugees may be less able to do so.

Our findings are consistent with studies on barriers to primary care for refugees in Canada. Studies have identified culturally inappropriate care, language barriers, and difficulty navigating the system as compounding barriers to care [24, 33]. Our health care providers reflected that it was important for them to understand their patient’s expectations of pregnancy and prenatal care in order to deliver optimal care tailored to their patient. Additionally, a lack of access to language services within the community made it difficult for health care professionals at the MRHC to refer patients to outside sources.

Our study highlights that one of the greatest strengths of the MRHC is that they are a clinic specialized in caring for refugees, and so are familiar with issues surrounding the IFHP and coverage of refugees. Central models of health care provision for refugees can also be found in Kitchener and Hamilton, Ontario [16, 17]. Both clinics, like the MRHC, consist of interdisciplinary teams and focus on coordinating the care of their patients within and outside of the clinics. A review of models of health care for refugees in Australia, the United States, Canada, Switzerland and the United Kingdom, found that successful models of care for refugees included interdisciplinary health care teams, coordination of care between health care professionals, and attention to both medical and social needs of patients [18]. Overall, central models of refugee health care have been found to be successful in providing comprehensive health care to newly arrived refugees, and help to mitigate some of the challenges that they face when trying to navigate and interact within the Canadian health care system [16–18].

MRHC, and similar interdisciplinary clinics specializing in refugee health, may be uniquely placed to understand the multiple sources of influence on the health of refugees. This intersectionality of influence recognizes that women’s health is influenced by their experiences in home countries, the migration experience and their experiences in a new host country, all of which are shaped by diverse perspectives on race, class, gender, and citizenship status [34]. While not explicitly stated as a theoretical perspective, health care providers in our study noted how the interdisciplinary nature of MRHC allowed them to support the physical needs of women, and their team of nurses, social workers and other allied professional enabled them to address the social needs of women as well. Because of the particular complexity and vulnerability of refugees’ situation, having health care providers who understand these influences is especially critical to providing care.

Few primary studies have yet to investigate the effect of the recent changes to the IFHP and Canada’s increased response efforts to the Syrian refugee crisis, on refugee health care providers. Several opinion pieces have been published assessing the potential impacts of the IFHP funding cuts. Harris & Zuberi [35] identified that pregnant women and children were specifically at risk following the funding cuts. As described by health care professionals, when women do not receive adequate prenatal care, they are at increased risk of delivering babies who may require additional care and resources to reach their potential. Therefore, the funding cuts adversely impact the health and wellbeing of Canadian children. Others have identified that the IFHP funding cuts resulted in confusion amongst health care professionals and added difficulty navigating the health care system [36, 37]. As explained by our participants, when additional strains are added to the health care system, it can make an already complicated and tedious system even more difficult to maintain high standards of care, and restrict access for an already vulnerable population.

In press coverage at the time of the cuts, the government stated that the purpose of the cuts was to save money (estimated at $100 million over five years), to protect public health and safety and to deter health tourism [38]. However, a 2014 SickKids Hospital Study in Toronto that was instrumental in reinstating IFHP coverage for pregnant women and children [39], concluded that the burden of costs was not reduced. SickKids Hospital has a policy of not turning away any patients, so were covering the expenses of uninsured patients out of pocket [39]. This is similar to how the MRHC developed alternative strategies to provide care to all patients regardless of their health care coverage status. When the cuts to the IFHP were declared unconstitutional in 2014, part of the ruling specified that the government had failed to provide evidence for any of the arguments made for the cuts [40]. Specifically, in her ruling, Justice Mactavish commented: “I would also note that there is a real question as to whether the cuts to the IFHP will in fact achieve any real cost savings to taxpayers — another stated objective of the changes — or whether the costs of providing medical care to those seeking the protection of Canada are simply being downloaded to the provinces and others” [40].

Few studies have yet to examine Canada’s response to the Syrian refugee crisis since November of 2015. However, testimonies from refugee health clinics and health care professionals in Canada indicate that they are struggling to manage the increased service demand since the beginning of the Syrian refugee influx, similar to the MRHC [41–43]. It is important to recognize that while the Canadian government allocated special resources to re-settling high numbers of Syrian refugees in Canada, they failed to provide additional support to the health care system that would be accommodating increased user demands. As identified by our participants, Syrian refugees have several unmet chronic health needs and many pregnant Syrian women have not received adequate prenatal care [42, 43]. This is especially noticeable in Syrian refugees because prior to the war, primary and reproductive health care was freely available and widely utilized in Syria. Now, many Syrian women have gone years without accessing any health care due to limited resources, restricted access, and prioritizing the health of other family members over themselves [44]. Further, chronic disease management and perinatal care is often not made a priority of humanitarian aid in conflict affected regions [45–47].

So despite the change in government, and the Liberal’s stated policy of increasing the number of Syrian refugees to Canada [48], the additional strain on health care providers has not been alleviated due to the rapid influx of a population with complex health needs. Dennis Raphael suggests that the Canadian approach is consistent with other Liberal welfare states (e.g. Australia, United Kingdom) in that the government is outwardly committed to providing the fundamental prerequisites for health but fails to fully match that commitment with actual policy and budgetary support [49]. To ensure equity in health care for all Canadians, support for vulnerable groups must be made available. This inherent belief in universal access to healthcare despite ability to pay is consistently reaffirmed by Canadians [50]. Several health care providers in our study spoke specifically to what it meant to be “Canadian”, which was rooted in a sense that everyone should have access to health care, and that if the government was not meeting this need, then conscious citizens would take on this burden.

Our study has limitations which must be considered. First, as is common to qualitative research, the generalizability of our study findings remains unknown, as they reflect the experiences of a small number of health care professionals (*n* = 10) in Calgary. However, we interviewed almost all of the professionals at MRHC who worked with pregnant refugees with the exception of the obstetricians at the MRHC due to scheduling constraints. We were only able to interview two participants from the hospital which limited comparisons between health care professionals at a clinic vs. a hospital. However, efforts to interview additional staff who did not work specifically with refugees was challenging due to lack of interest and lack of knowledge about the topics covered in the interview. Despite our small sample, our findings are consistent with previous studies which have found that prior to the IFHP funding cuts, Canadian health care professionals struggled to care for refugee patients because of lack of understanding as to how the IFHP works [36, 37]. Consequently, several primary care centres in Canada do not accept patients covered by the IFHP or direct bill them [36, 37, 51].

Our study has several strengths. One key strength was that our participants were highly qualified to speak to the phenomenon under study. Also, while mentioned in limitations, having two participants from the hospital was also a strength, as it expanded our study sample reach beyond a single location. Furthermore, our study sample contained a variety of health care professionals, which allowed us to get multiple perspectives on the same questions. In general, we found that all participants had very similar views on issues and identified the same barriers, strategies and needs for both health care professionals and patients. Our member checking process did support that we had achieved thematic saturation. Given this, we believe that we achieved thematic saturation by the end of the 10 interviews.

## Conclusion

In 2017, Canada is assisting with the global refugee crisis by accepting more refugees into the country than ever before. It was critical to assess the needs of health care professionals providing frontline care to refugee patients of whom a large proportion required perinatal care. We highlighted five main findings. First, pregnant refugees are a distinct and vulnerable population, due to the time-sensitive nature of perinatal care. Proper pregnancy care can be compromised by the multitude of barriers that health care professionals and refugee patients face, potentially impacting pregnancy and child outcomes. Second, health care professionals specialized in refugee health recognize that pregnancy is often an entry point into the Canadian HCS and engage in diverse strategies to optimize care for mother and baby’s health. Next, additional system strains on an already overburdened HCS, such as the 2012–2016 funding cuts to refugee health care and recent Syrian refugee influx, jeopardize patient health and require extraordinary efforts by health care professionals to maintain patient quality of care. Finally, health care professionals not trained in refugee health may struggle to navigate the system and provide optimal care to refugee patients. In general, working in the field of refugee health care may be more demanding and challenging than other fields of health care, and therefore it takes service providers with certain characteristics to specialize in refugee health.

### Recommendations

Through discussion with our participants, as well as other staff members at the MRHC, we have developed three recommendations to improve the provision of health care for refugees in Canada:

1. A central clinic model for providing health care for refugees can help ensure that relevant expertise is concentrated, and refugees receive appropriate care while adapting to a complex system in a new environment.
2. The current system for health care provision for refugees is complicated, tedious and not well understood by many health care professionals. Initiatives should be taken to make the system more streamlined and accessible to all service providers.
3. Increased financial support to clinics that serve the needs of refugees would enable comprehensive health care services to improve outcomes for vulnerable populations.

## Additional file

Additional file 1: Coding Framework. (XLSX 16 kb)

## Abbreviations

IFHP: Interim Federal Health Program
MRHC: Mosaic Refugee Health Clinic
